# Supplementary material for: Adoption of a biologically-enhanced agricultural management (BEAM) approach in agroecosystems for regenerating soil fertility, improving farm profitability and achieving productive utilization of atmospheric CO2
Source: PeerJ. 2025 Mar 31;13:e19167. doi: 10.7717/peerj.19167 (PMC11967414; doi:10.7717/peerj.19167)

Figure S-1: Research was conducted on this 5.22-hectare field plot (Lat. 37.779322°, Lon. 27.494952°), 8.5 kilometers from Soke, Turkey. Treatment borders are outlined in red and sample points are designated within treatment borders.

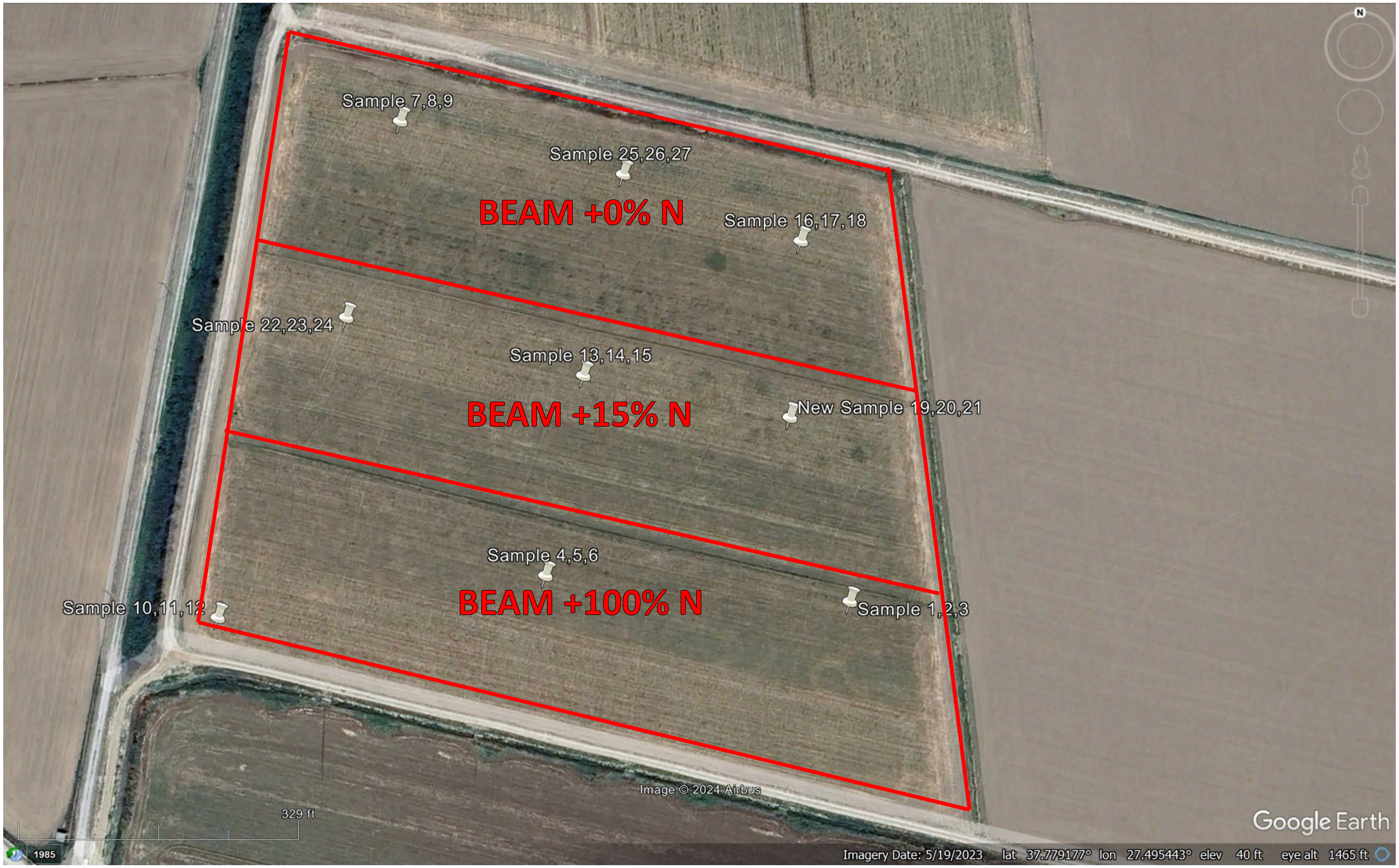

Supplement: Supplemental Information 1 — Research was conducted on this 5.22-hectare field plot (Lat. 37.779322°, Lon. 27.494952°), 8.5 kilometers from Soke, Turkey within treatment borders and associated sample GPS locations. Image courtesy of Google Earth Pro, Image © 2024 Airbus. [file peerj-13-19167-s001.pdf]
