# Supplementary material for: Adoption of a biologically-enhanced agricultural management (BEAM) approach in agroecosystems for regenerating soil fertility, improving farm profitability and achieving productive utilization of atmospheric CO2
Source: PeerJ. 2025 Mar 31;13:e19167. doi: 10.7717/peerj.19167 (PMC11967414; doi:10.7717/peerj.19167)

Figure S-2 Two-sample ANOVA statistical analysis of Soil Organic Carbon (%) comparing the three fertilizer treatments (100% N, 15% N and 0% N) administered for cotton production, to observe fertilization rate influence on 2023 soil organic carbon% at each of the three depths (0-15 cm, 15-30 cm and 30-45 cm).

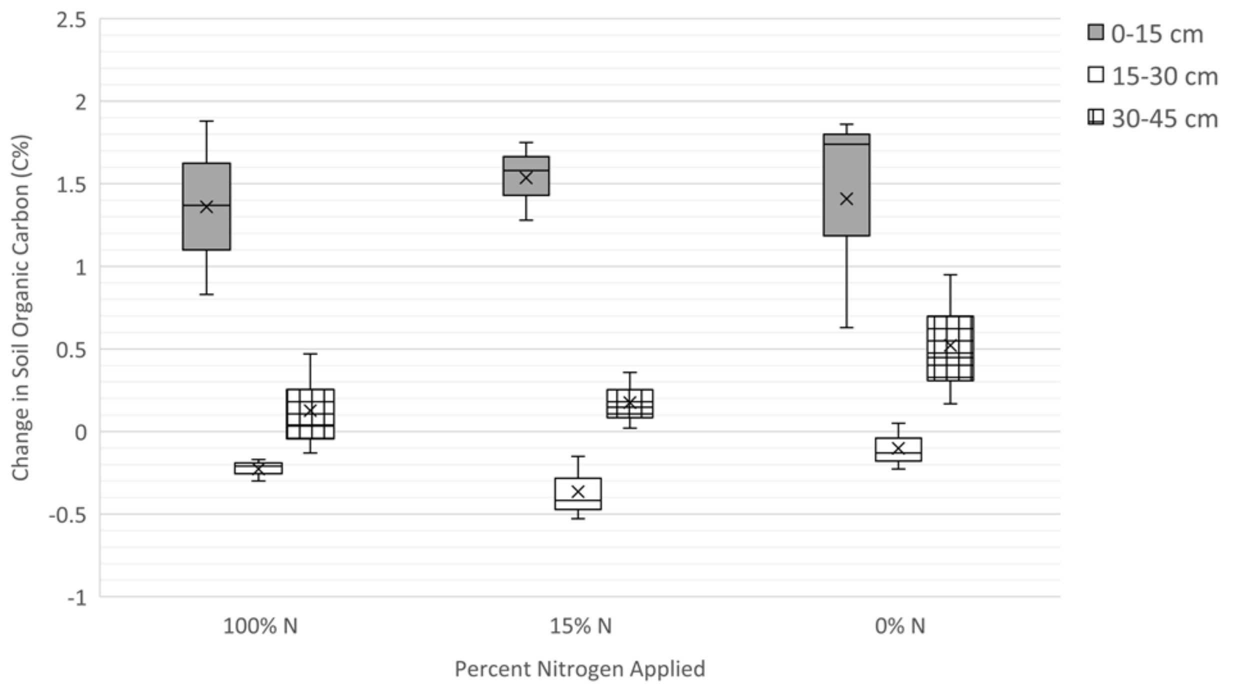

Supplement: Supplemental Information 2 — Two-sample ANOVA statistical analysis, comparing the three fertilizer treatments (100% N, 15% N and 0% N) administered for cotton production, to observe it’s potential influence on 2023 SOC% at each of the three soil depths (0–15 cm, 15–30 cm and 30–45 cm). [file peerj-13-19167-s002.pdf]
