# Supplementary material for: Adoption of a biologically-enhanced agricultural management (BEAM) approach in agroecosystems for regenerating soil fertility, improving farm profitability and achieving productive utilization of atmospheric CO2
Source: PeerJ. 2025 Mar 31;13:e19167. doi: 10.7717/peerj.19167 (PMC11967414; doi:10.7717/peerj.19167)

Figure S-3 Two-sample ANOVA statistical analysis of total soil nitrogen percent (TSN%) comparing the three fertilizer treatments (100% N, 15% N and 0% N) administered for cotton production, to observe fertilization rate influence on 2023 TSN% at each of the three depths (0-15 cm, 15-30 cm and 30-45 cm).

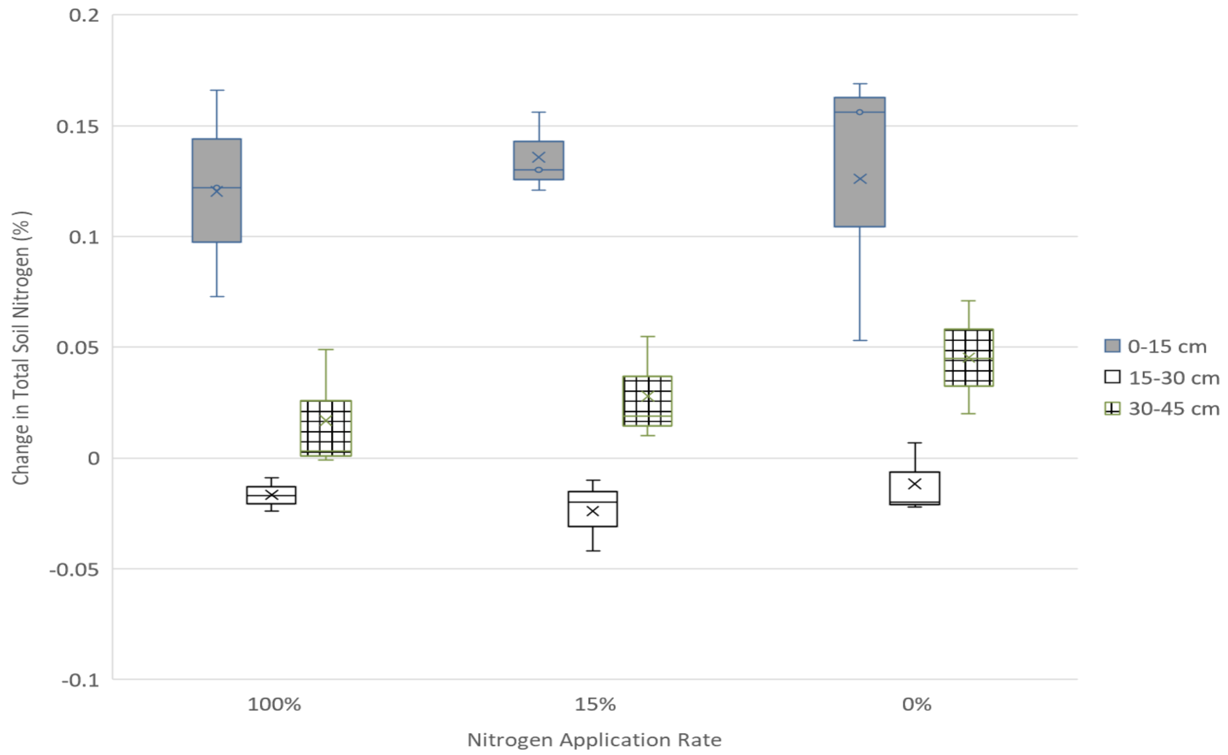

Supplement: Supplemental Information 3 — Two-sample ANOVA statistical analysis comparing the three fertilizer treatments (100% N, 15% N and 0% N) administered for cotton production, to observe its potential influence on 2023 TSN% at each of the three depths (0–15 cm, 15–30 cm and 30–45 cm). [file peerj-13-19167-s003.pdf]
