# Supplementary material for: Adoption of a biologically-enhanced agricultural management (BEAM) approach in agroecosystems for regenerating soil fertility, improving farm profitability and achieving productive utilization of atmospheric CO2
Source: PeerJ. 2025 Mar 31;13:e19167. doi: 10.7717/peerj.19167 (PMC11967414; doi:10.7717/peerj.19167)
Supplement: Supplemental Information 4 [file peerj-13-19167-s004.docx]

Table S-1 Multi-species summer and winter cover crop seed varieties and application rates for 2019, 2019-2020, 2020-2021, 2021-2022, and 2022-2023 cover crops.

| **2019 Summer** | | |
| --- | --- | --- |
| Name | Pounds ac^-1^ | kg ha^-1^ |
| Hairy Vetch (*Vicia villosa*) | 9 | 10 |
| Peas: Austrian (*Pisum sativum subsp. Arvense*) | 4.55 | 5 |
| Triticale *hexaploide Lart* | 4 | 4 |
| Nitro radish (*Raphanus raphanistrum subsp. sativus*) | 0.73 | 0.82 |
| Phacelia (*Phacelia tanacetifolia*) | 1.09 | 1.22 |
| Buckwheat (*Fagopyrum esculentum*) | 2.73 | 3.07 |
| Oil Sunflower (*Helianthus annuus*) | 5 | 5.61 |
| Sugar beet | 1.5 | 1.68 |
| Sorghum | 25 | 28.07 |
| **2019-2020 -Winter** | | |
| Name | Pounds ac^-1^ | kg ha^-1^ |
| Hairy Vetch (*Vicia villosa*) | 6 | 7 |
| Peas: Austrian (*Pisum sativum subsp. Arvense*) | 26 | 29 |
| Hairy Vetch (*Vicia villosa*) | 10 | 11 |
| Triticale *hexaploide Lart* | 10.94 | 12.28 |
| Nitro radish (*Raphanus raphanistrum subsp. sativus*) | 3.12 | 3.50 |
| Turnip | 1.04 | 1.17 |
| Sugar beet | 0.91 | 1.02 |
| **2020-2021 Winter** | | |
| Name | Pounds ac^-1^ | kg ha^-1^ |
| Hairy Vetch (*Vicia villosa*) | 16 | 18 |
| Peas: Austrian (*Pisum sativum subsp. Arvense*) | 26 | 29 |
| Triticale *hexaploide Lart* | 10.94 | 12 |
| Nitro radish (*Raphanus raphanistrum subsp. sativus*) | 6 | 6.74 |
| **2021-2022 Winter** | | |
| Name | Pounds ac^-1^ | kg ha^-1^ |
| Hairy Vetch (*Vicia villosa*) | 16 | 18 |
| Peas: Austrian (*Pisum sativum subsp. Arvense*) | 26 | 29 |
| Rye | 10.94 | 12 |
| Nitro radish (*Raphanus raphanistrum subsp. sativus*) | 6 | 6.74 |
|  |  |  |
| **2022-2023 Winter** | | |
| Name | Pounds ac^-1^ | kg ha^-1^ |
| Hairy Vetch (*Vicia villosa*) | 16 | 18 |
| Peas: Austrian (*Pisum sativum subsp. Arvense*) | 26 | 29 |
| Rye | 10.94 | 12 |
| Nitro radish (*Raphanus raphanistrum subsp. sativus*) | 6 | 6.74 |
|  |  |  |
