# Supplementary material for: Adoption of a biologically-enhanced agricultural management (BEAM) approach in agroecosystems for regenerating soil fertility, improving farm profitability and achieving productive utilization of atmospheric CO2
Source: PeerJ. 2025 Mar 31;13:e19167. doi: 10.7717/peerj.19167 (PMC11967414; doi:10.7717/peerj.19167)
Supplement: Supplemental Information 5 [file peerj-13-19167-s005.docx]

Table S-2 Treatment sample data for sample sites, soil depth, fertilizer treatments, bulk density, soil organic carbon% (SOC%) and total soil nitrogen% (TSN%) for years 2019, 2020, 2022, and 2023.

| ­­­­­­­­ | Coordinates | Depth (cm) | Fertilizer Treatment | Sample No. | Bulk Density | Soil Organic Carbon (%) | | | |  | Total Soil Nitrogen (%) | | | |
| --- | --- | --- | --- | --- | --- | --- | --- | --- | --- | --- | --- | --- | --- | --- |
|  |  |  |  |  |  | **2019** | **2020** | **2022** | **2023** |  | **2019** | **2020** | **2022** | **2023** |
| Sample  1-2-3 | N 37°46'42,98988" E 27°29'45,80916" | 0-15 | 100% N | 1 | 1.53 | 0.44 | 1.14 | 1.01 | 1.27 |  | 0.03 | 0.056 | 0.09 | 0.103 |
|  |  | 15-30 |  | 2 | 1.57 | 1.07 | 0.88 | 0.55 | 0.77 |  | 0.09 | 0.046 | 0.05 | 0.066 |
|  |  | 30-45 |  | 3 | 1.49 | 0.58 | 0.45 | 0.29 | 0.62 |  | 0.05 | 0.045 | 0.02 | 0.053 |
| Sample  4-5-6 | N 37°46'43,29408" E 27°29'41,36748" | 0-15 | 100% N | 4 | 1.62 | 0.28 | 0.73 | 1.15 | 2.16 |  | 0.02 | 0.05 | 0.1 | 0.186 |
|  |  | 15-30 |  | 5 | 1.52 | 0.76 | 0.49 | 0.65 | 0.59 |  | 0.06 | 0.04 | 0.06 | 0.051 |
|  |  | 30-45 |  | 6 | 1.83 | 0.41 | 0.35 | 0.59 | 0.88 |  | 0.03 | 0.04 | 0.05 | 0.079 |
| Sample  7-8-9 | N 37°46'48,44568" E 27°29'39,15528" | 0-15 | 0% N | 7 | 1.52 | 0.42 | 1.14 | 1.45 | 2.28 |  | 0.03 | 0.1 | 0.12 | 0.197 |
|  |  | 15-30 |  | 8 | 1.85 | 0.74 | 0.66 | 0.94 | 0.61 |  | 0.06 | 0.04 | 0.08 | 0.05 |
|  |  | 30-45 |  | 9 | 1.45 | 0.31 | 0.64 | 0.86 | 1.26 |  | 0.02 | 0.04 | 0.07 | 0.109 |
| Sample  10-11-12 | N 37°46'42,76812" E 27°29'36,49812" | 0-15 | 100% N | 10 | 1.67 | 0.28 | 0.62 | 1.23 | 1.65 |  | 0.02 | 0.04 | 0.11 | 0.142 |
|  |  | 15-30 |  | 11 | 1.57 | 0.82 | 0.34 | 0.62 | 0.61 |  | 0.07 | 0.02 | 0.05 | 0.053 |
|  |  | 30-45 |  | 12 | 1.5 | 0.58 | 0.17 | 0.72 | 0.45 |  | 0.04 | 0.01 | 0.06 | 0.039 |
| Sample 13-14-15 | N 37°46'45,57072" E 27°29'41,83872" | 0-15 | 15% N | 13 | 1.56 | 0.53 | 0.77 | 1.77 | 1.81 |  | 0.04 | 0.04 | 0.15 | 0.161 |
|  |  | 15-30 |  | 14 | 1.63 | 0.96 | 0.67 | 1.21 | 0.81 |  | 0.08 | 0.03 | 0.1 | 0.07 |
|  |  | 30-45 |  | 15 | 1.49 | 0.68 | 0.66 | 0.73 | 0.7 |  | 0.05 | 0.03 | 0.06 | 0.06 |
| Sample  16-17-18 | N 37°46'47,63208" E 27°29'45,74256" | 0-15 | 0% N | 16 | 1.45 | 0.64 | 0.96 | 0.99 | 2.13 |  | 0.05 | 0.04 | 0.09 | 0.184 |
|  |  | 15-30 |  | 17 | 1.59 | 1.8 | 0.46 | 0.5 | 0.92 |  | 0.15 | 0.02 | 0.04 | 0.079 |
|  |  | 30-45 |  | 18 | 1.55 | 1.03 | 0.86 | 1.12 | 0.68 |  | 0.09 | 0.04 | 0.01 | 0.058 |
| Sample 19-20-21 | N 37°46'45,0282" E 27°29'44,7666" | 0-15 | 15% N | 19 |  |  |  |  | 1.97 |  |  |  |  | 0.16 |
|  |  | 15-30 |  | 20 |  |  |  |  | 0.46 |  |  |  |  | 0.04 |
|  |  | 30-45 |  | 21 |  |  |  |  | 0.87 |  |  |  |  | 0.075 |
| Sample 22-23-24 | N 37°46'46,17192" E 27°29'38,16348" | 0-15 | 15% N | 22 |  |  |  |  | 2.14 |  |  |  |  | 0.184 |
|  |  | 15-30 |  | 23 |  |  |  |  | 0.35 |  |  |  |  | 0.03 |
|  |  | 30-45 |  | 24 |  |  |  |  | 0.66 |  |  |  |  | 0.057 |
| Sample 25-26-27 | N 37°46'47,88588" E 27°29'42,36" | 0-15 | 0% N | 25 |  |  |  |  | 1.02 |  |  |  |  | 0.081 |
|  |  | 15-30 |  | 26 |  |  |  |  | 0.65 |  |  |  |  | 0.052 |
|  |  | 30-45 |  | 27 |  |  |  |  | 0.96 |  |  |  |  | 0.083 |
