# Supplementary material for: Adoption of a biologically-enhanced agricultural management (BEAM) approach in agroecosystems for regenerating soil fertility, improving farm profitability and achieving productive utilization of atmospheric CO2
Source: PeerJ. 2025 Mar 31;13:e19167. doi: 10.7717/peerj.19167 (PMC11967414; doi:10.7717/peerj.19167)
Supplement: Supplemental Information 6 [file peerj-13-19167-s006.docx]

|  |  | Yr | 2019 | 2020 | 2021 | 2022 | 2023 |  |  |  |  |  |  |
| --- | --- | --- | --- | --- | --- | --- | --- | --- | --- | --- | --- | --- | --- |
|  |  | $/gal | $ 3.06 | $ 2.55 | $ 3.29 | $ 4.99 | $ 4.21 |  |  |  |  |  |  |
|  | Usage gal/ac | Usage gal/ha |  |  |  |  |  |  |  |  |  |  |  |
| Moldboard | 1.85 | 4.57 | $ 13.96 | $ 11.65 | $ 15.02 | $ 22.80 | $ 19.26 |  |  |  |  |  |  |
| Chisel | 1.25 | 3.09 | $ 9.44 | $ 7.87 | $ 10.15 | $ 15.40 | $ 13.01 |  |  |  |  |  |  |
| Discing | 0.6 | 1.48 | $ 4.53 | $ 3.78 | $ 4.87 | $ 7.39 | $ 6.25 |  |  |  |  |  |  |
| Cultivating | 0.6 | 1.48 | $ 4.53 | $ 3.78 | $ 4.87 | $ 7.39 | $ 6.25 |  |  |  |  |  |  |
| Fertilizing | 0.7 | 1.73 | $ 5.28 | $ 4.41 | $ 5.68 | $ 8.63 | $ 7.29 |  |  |  |  |  |  |
| Planting | 0.95 | 2.35 | $ 7.17 | $ 5.98 | $ 7.71 | $ 11.71 | $ 9.89 |  |  |  |  |  |  |
| Cultivating (Sweeps) | 0.35 | 0.86 | $ 2.64 | $ 2.20 | $ 2.84 | $ 4.31 | $ 3.64 |  |  |  |  |  |  |
| Spraying | 0.15 | 0.37 | $ 1.13 | $ 0.94 | $ 1.22 | $ 1.85 | $ 1.56 |  |  |  |  |  |  |
|  |  |  | **Conventional** | | | | |  | **Biologically Enhanced Agricultural Management** | | | | |
| 1 | Deep cultivator |  | $ 13.96 | $ 11.65 | $ 15.02 | $ 22.80 | $ 19.26 |  |  |  |  |  |  |
| 2 | Fertilizer |  | $ 5.28 | $ 4.41 | $ 5.68 | $ 8.63 | $ 7.29 |  | $ 5.28 | $ 4.41 | $ 5.68 | $ 8.63 | $ 7.29 |
| 3 | Fertilizer |  | $ 5.28 | $ 4.41 | $ 5.68 | $ 8.63 | $ 7.29 |  |  |  |  |  |  |
| 4 | Disc Harrow |  | $ 4.53 | $ 3.78 | $ 4.87 | $ 7.39 | $ 6.25 |  |  |  |  |  |  |
| 5 | Cultivator |  | $ 4.53 | $ 3.78 | $ 4.87 | $ 7.39 | $ 6.25 |  |  |  |  |  |  |
| 6 | Harrow |  | $ 4.53 | $ 3.78 | $ 4.87 | $ 7.39 | $ 6.25 |  |  |  |  |  |  |
| 7 | Herbicide |  | $ 1.13 | $ 0.94 | $ 1.22 | $ 1.85 | $ 1.56 |  |  |  |  |  |  |
| 8 | Seed Drill |  | $ 7.17 | $ 5.98 | $ 7.71 | $ 11.71 | $ 9.89 |  | $ 7.17 | $ 5.98 | $ 7.71 | $ 11.71 | $ 9.89 |
| 9 | Hebicide |  | $ 1.13 | $ 0.94 | $ 1.22 | $ 1.85 | $ 1.56 |  |  |  |  |  |  |
| 10 | Pesticide |  | $ 1.13 | $ 0.94 | $ 1.22 | $ 1.85 | $ 1.56 |  | $ 1.13 | $ 0.94 | $ 1.22 | $ 1.85 | $ 1.56 |
| 11 | Insecticide |  | $ 1.13 | $ 0.94 | $ 1.22 | $ 1.85 | $ 1.56 |  | $ 1.13 | $ 0.94 | $ 1.22 | $ 1.85 | $ 1.56 |
| 12 | Insecticide |  | $ 1.13 | $ 0.94 | $ 1.22 | $ 1.85 | $ 1.56 |  | $ 1.13 | $ 0.94 | $ 1.22 | $ 1.85 | $ 1.56 |
| 13 | Insecticide |  | $ 1.13 | $ 0.94 | $ 1.22 | $ 1.85 | $ 1.56 |  | $ 1.13 | $ 0.94 | $ 1.22 | $ 1.85 | $ 1.56 |
| 14 | Fertilizer |  | $ 5.28 | $ 4.41 | $ 5.68 | $ 8.63 | $ 7.29 |  |  |  |  |  |  |
| 15 | Fertilizer |  | $ 5.28 | $ 4.41 | $ 5.68 | $ 8.63 | $ 7.29 |  |  |  |  |  |  |
| 16 | Foliar Nutrition |  | $ 1.13 | $ 0.94 | $ 1.22 | $ 1.85 | $ 1.56 |  | $ 1.13 | $ 0.94 | $ 1.22 | $ 1.85 | $ 1.56 |
| 17 | Foliar Nutrition |  | $ 1.13 | $ 0.94 | $ 1.22 | $ 1.85 | $ 1.56 |  | $ 1.13 | $ 0.94 | $ 1.22 | $ 1.85 | $ 1.56 |
| 18 | Herbicide |  | $ 1.13 | $ 0.94 | $ 1.22 | $ 1.85 | $ 1.56 |  |  |  |  |  |  |
| 20 | Foliar Nutrition |  | $ 1.13 | $ 0.94 | $ 1.22 | $ 1.85 | $ 1.56 |  | $ 1.13 | $ 0.94 | $ 1.22 | $ 1.85 | $ 1.56 |
| 22 | Foliar Nutrition |  | $ 1.13 | $ 0.94 | $ 1.22 | $ 1.85 | $ 1.56 |  | $ 1.13 | $ 0.94 | $ 1.22 | $ 1.85 | $ 1.56 |
| 24 | Defoliation |  | $ 1.13 | $ 0.94 | $ 1.22 | $ 1.85 | $ 1.56 |  | $ 1.13 | $ 0.94 | $ 1.22 | $ 1.85 | $ 1.56 |
| 26 | Mulching |  | $ 4.53 | $ 3.78 | $ 4.87 | $ 7.39 | $ 6.25 | Roll Down | $ 2.64 | $ 2.20 | $ 2.84 | $ 4.31 | $ 3.64 |
|  |  |  | $ 73.97 | $ 61.73 | $ 79.57 | $ 120.76 | $ 102.00 |  | $ 25.29 | $ 21.10 | $ 27.20 | $ 41.28 | $ 34.87 |
|  |  |  |  |  |  | Annual Fuel Cost | **$ 438.03** |  |  |  |  | Annual Fuel Cost | **$ 149.74** |

Table S-3 Fuel cost estimates for farm practices comparing a biologically enhanced agricultural management to conventional practices.
