# Supplementary material for: Adoption of a biologically-enhanced agricultural management (BEAM) approach in agroecosystems for regenerating soil fertility, improving farm profitability and achieving productive utilization of atmospheric CO2
Source: PeerJ. 2025 Mar 31;13:e19167. doi: 10.7717/peerj.19167 (PMC11967414; doi:10.7717/peerj.19167)
Supplement: Supplemental Information 7 [file peerj-13-19167-s007.docx]

| HERBICIDES | Application Rate | | $/g | Cost/ha |
| --- | --- | --- | --- | --- |
| Stomp | 3000 | g/ha | 0.007 | $ 21.00 |
| Syngenta Envoke | 14 | g/ha | 4.59 | $ 64.26 |
| Clethodim | 750 | g/ha | 0.014 | $ 10.50 |
| Tractor Cost $/ha/trip |  | $ 19.76 | 3 | $ 59.28 |
|  |  |  | Total | $ 155.04 |
|  |  | % Reduction | 100% |  |
|  |  |  |  |  |
|  |  |  |  |  |
|  |  |  |  |  |
| PESTIDICES/INSECTICIDES | Application Rate | | $/g | Cost/ha |
| Abamectin | 400 | g/ha | 0.019 | $ 7.60 |
| Dimethoate | 600 | g/ha | 0.045 | $ 27.00 |
| Imadacloprid + Beta Cyflurthin | 200 | g/ha | 0.022 | $ 4.40 |
| Ememactin Benzoate | 400 | g/ha | 0.07 | $ 28.00 |
| Tractor Cost $/ha/trip |  | $ 19.76 | 4 | $ 79.04 |
|  |  |  | Total | $ 146.04 |
|  |  | 65% Reduction | 56% | $ 81.78 |
|  |  |  |  |  |
|  |  |  | Conventional | $ 301.08 |
|  |  |  | BEAM | $ 81.78 |

Table S-4 Herbicide and Pesticide/Insecticide cost estimates comparing a biologically enhanced agricultural management approach to a conventional approach.
